# Accuracy and Timeliness of Prehospital Global Triage System Protocols in Mass Disasters: A Systematic Review of Systematic Reviews

**DOI:** 10.7759/cureus.92796

**Published:** 2025-09-20

**Authors:** Amr Essam Shaltout, Mohammed Elfatih Elbadri, Kiranjot Kaur, Mohammed M Alsharif, Aliaa H. Alkhazendar, Omar Nasr Hassouba, Muhammad Nabeel Ahmad, Mazin Osman, Areeba Zahid, Shaun Banjamin

**Affiliations:** 1 Emergency Department, Nottingham University Hospital, Nottingham, GBR; 2 Department of Orthopedics, Khorfakkan Hospital, Emirates Health Services, Sharjah, ARE; 3 Department of Medicine, United States Navy, United States Military, North Chicago, USA; 4 Department of Clinical Research, Arizona State University, Tempe, USA; 5 Department of Internal Medicine, Shri B. M. Patil Medical College, Bijapur, IND; 6 Department of General Surgery, Al Ahli Hospital, Hebron, PSE; 7 Department of Surgery, The Islamic University of Gaza, Gaza, PSE; 8 Emergency Department, The Shrewsbury and Telford Hospital NHS Trust, Shrewsbury, GBR; 9 Department of Internal Medicine, University Hospitals of Morecambe Bay NHS Foundation Trust, Lancaster, GBR; 10 Department of General Surgery, British United Provident Association, Jeddah, SAU; 11 Department of Internal Medicine, Faisalabad Medical University and Hospital, Faisalabad, PAK; 12 Department of General Medicine, Dow University of Health Sciences, Karachi, PAK

**Keywords:** artificial intelligence, jumpstart, mass casualty incidents, modified physiological triage tool (mptt), prehospital triage, salt, start

## Abstract

This systematic review evaluated the accuracy and timeliness of global prehospital triage systems in mass disasters, following Preferred Reporting Items for Systematic reviews and Meta-Analyses (PRISMA) 2020 guidelines. A comprehensive search of PubMed/MEDLINE, Embase, Scopus, and Cochrane Library up to June 2025 identified 344 records, of which four studies met eligibility criteria after screening and full-text assessment. Included studies analyzed conventional systems such as Simple Triage and Rapid Treatment (START), JumpSTART, Sort, Assess, Lifesaving Interventions, Treatment/Transport (SALT), and Modified Physiological Triage Tool (MPTT), as well as artificial intelligence (AI)-assisted approaches and diagnostic adjuncts like portable ultrasound. Sample sizes ranged from targeted reviews of 30-60 studies (systematic and evidence-based reviews) to practical evaluations of triage innovations involving prehospital and emergency responders. Data extraction captured accuracy, timeliness, and resource allocation, while risk of bias was assessed using the A Measurement Tool to Assess Systematic Reviews version 2 (AMSTAR-2) and the Scale for the Assessment of Narrative Review Articles (SANRA), with ratings ranging from low to moderate. Results demonstrated that traditional systems such as START and SALT provide rapid categorization but remain prone to over- and under-triage depending on responder training and situational factors. AI-driven models and portable diagnostic technologies significantly improved decision speed, diagnostic precision, and prioritization of life-saving interventions, reducing delays in critical care. Overall, while no single algorithm proved universally superior, integration of training, simulation-based preparedness, and emerging AI-supported tools was consistently associated with improved triage performance in chaotic, resource-limited disaster environments.

## Introduction and background

Triage, in common man terms, means “to sort.” It is the rapid clinical process of prioritizing multiple patients when needs exceed immediately available resources. In emergency care, this means assigning patients to categories that reflect the urgency of intervention so that limited time, personnel, and equipment produce the greatest overall survival. The science of triage hinges on two intertwined performance attributes: accuracy, which is how well categories reflect the true need for life-saving intervention, and timeliness, representing how quickly correct decisions are made. In practice, both are measured against outcomes such as need for life-saving interventions, critical care, or mortality, and expressed with diagnostic metrics including sensitivity, specificity, over-triage, and under-triage to quantify the risks of misclassification at the scene. Decades of research have shown that excessive over-triage can flood downstream facilities, while under-triage can delay definitive care for the sickest patients, each with real mortality costs. More recently, systematic evaluations and meta-analyses have operationalized these diagnostic concepts for disaster triage algorithms, strengthening the evidence base for what works and what doesn’t when seconds matter [1].

Against this clinical backdrop, mass disasters introduce a scale and complexity that stress even well-designed systems: chaotic scenes, compromised communications, variable responder experience, and heterogeneous injury patterns. To bring order to this chaos, multiple prehospital algorithms have been developed and studied across settings. The most common of which are Simple Triage and Rapid Treatment (START), Modified START (mSTART), Sort, Assess, Lifesaving Interventions, Treatment/Transport (SALT), Smart, CareFlight, Amberg-Schwandorf Algorithm for Primary Triage (ASAV), Modified Physiological Triage Tool (MPTT), Sieve (Triage Sieve), and the hospital-based Emergency Severity Index (ESI) triage system [2]. Each operationalizes a slightly different balance of speed versus precision using simple physiology (e.g., respiratory rate), perfusion surrogates, mental status, observable behavior (e.g., ability to walk), or structured life-saving interventions embedded in the algorithm. SALT emerged from a national guideline effort to harmonize nomenclature and steps for mass-casualty triage and has demonstrated good usability and speed in simulations [3]. START remains widely taught and used, with real-world assessments showing pragmatic performance but also highlighting risks of both over- and under-triage depending on context [4]. CareFlight and Triage Sieve have been compared head-to-head with START, with data suggesting comparatively lower diagnostic performance for Sieve in predicting severe injury or life-saving intervention.

In the last decade, physiology-driven tools such as the MPTT and its update (MPTT-24) were derived from large trauma datasets to maximize sensitivity for life-saving intervention while keeping under-triage low, which is an explicit attempt to ground primary triage in empirically optimized thresholds [5]. European systems have also innovated: the ASAV was purpose-built for physician-manned EMS and has been prospectively examined for diagnostic reliability, sensitivity, specificity, time to triage, and comprehensive performance, with refresher training shown to sustain accuracy over time [6]. Beyond primary scene triage, the ESI, a five-level resource-prediction instrument which structures emergency department flow and is sometimes adapted for secondary triage after initial field categorization, has substantial validation literature and ongoing quality improvement work addressing mistriage and equity. Collectively, this ecosystem illustrates why no single algorithm is universally superior across all incidents: incident type, casualty mix (adult, pediatric), provider training, and system constraints can shift the preferred balance between speed and diagnostic discrimination.

Mass casualty incidents (MCIs) present a uniquely challenging operational environment, where responders must rapidly identify and prioritize patients based on injury severity, available resources, and evolving situational. Triage systems, such as START, JumpSTART, SALT, CareFlight, and Triage Sieve, have been widely adopted to guide these decisions, each balancing speed against diagnostics. While comparative studies demonstrate variability in sensitivity, specificity, and rates of over- and under-triage, performance is also shaped by responder training, incident type, and environmental constraints [7]. Understanding these nuances is critical for optimizing triage protocols, improving patient outcomes, and ensuring efficient resource allocation in high-stakes, resource-limited scenarios. This systematic review aims to evaluate the accuracy and timeliness of global prehospital triage systems used in mass disaster scenarios, with a focus on identifying factors influencing their real-world performance and potential strategies for optimization.

## Review

Materials and methods

Search Strategy

This systematic review adheres to the Preferred Reporting Items for Systematic Reviews and Meta-Analyses (PRISMA) 2020 guidelines to ensure methodological rigor and transparency [8]. A comprehensive literature search was conducted in four major biomedical databases: PubMed/MEDLINE, Embase, Scopus, and the Cochrane Library. The search strategy employed a combination of medical subject headings (MeSH) and keywords such as “prehospital triage”, “mass casualty incidents”, “START”, “JumpSTART”, and “artificial intelligence”. Boolean operators were utilized to optimize sensitivity and specificity. The search was limited to human studies published in English up to June 2025.

Protocol Registration

This review was not registered in PROSPERO or any other systematic review protocol database [9]. Nonetheless, the methodology (eligibility criteria, search strategy, data extraction, and synthesis approach) was established a priori and conducted according to the PRISMA 2020 guidelines to ensure transparency and minimize bias.

Eligibility Criteria

This review included studies that met specific population, intervention, comparator, and outcome (PICO) criteria, focusing on patients involved in MCIs requiring prehospital triage, where interventions involved the implementation of prehospital triage systems, including both traditional and AI-assisted protocols, and comparisons assessed performance metrics such as accuracy, timeliness, and resource allocation, with outcomes evaluating the effectiveness of these systems in real-world disaster scenarios [10]. Inclusion criteria encompassed studies evaluating prehospital triage systems in mass casualty events, emphasizing accuracy and timeliness of protocols, involving human subjects, published in English between 2010 and 2025, peer-reviewed, and providing quantitative data on performance. Exclusion criteria ruled out animal studies, case reports, and editorials, as well as research that did not assess triage system performance, non-peer-reviewed articles, studies not in English, and those focusing solely on hospital-based triage systems.

Study Selection

Two reviewers independently screened the titles and abstracts of identified records for relevance. Full-text articles of potentially eligible studies were then assessed for inclusion. Discrepancies were resolved through discussion or consultation with a third reviewer. A PRISMA flow diagram was used to document the selection process.

Data Extraction

Data were independently extracted by two reviewers using a standardized, pre-piloted extraction form to ensure methodological consistency and minimize bias. The extracted variables encompassed detailed study characteristics (author, year, design, setting, and population), the specific prehospital triage system evaluated, and key performance indicators including accuracy, timeliness, and resource allocation efficiency. Additional outcome measures, such as mortality rates, morbidity, and overall system effectiveness in mass casualty contexts, were also recorded. In alignment with best research practices, any discrepancies between reviewers were addressed through structured discussion to achieve consensus, and when necessary, arbitration by a third senior reviewer was sought to resolve persistent disagreements, thereby ensuring data reliability and validity.

Risk of Bias Assessment

The risk of bias in the included studies was assessed using the A Measurement Tool to Assess Systematic Reviews version 2 (AMSTAR-2) for systematic reviews [11]. The Scale for the Assessment of Narrative Review Articles (SANRA) was used for non-randomized studies [12]. These tools evaluate potential biases in areas such as selection, performance, detection, and reporting. The assessments were conducted independently by two reviewers, with discrepancies resolved through discussion.

Data Synthesis

Due to the heterogeneity of the included studies, a qualitative synthesis was performed. Findings were categorized based on triage system type (traditional vs. AI-assisted), performance metrics, and outcomes. Trends and patterns were identified to provide a comprehensive overview of the effectiveness of prehospital triage systems in MCIs.

Results

Study Selection Process

**Figure 1:**
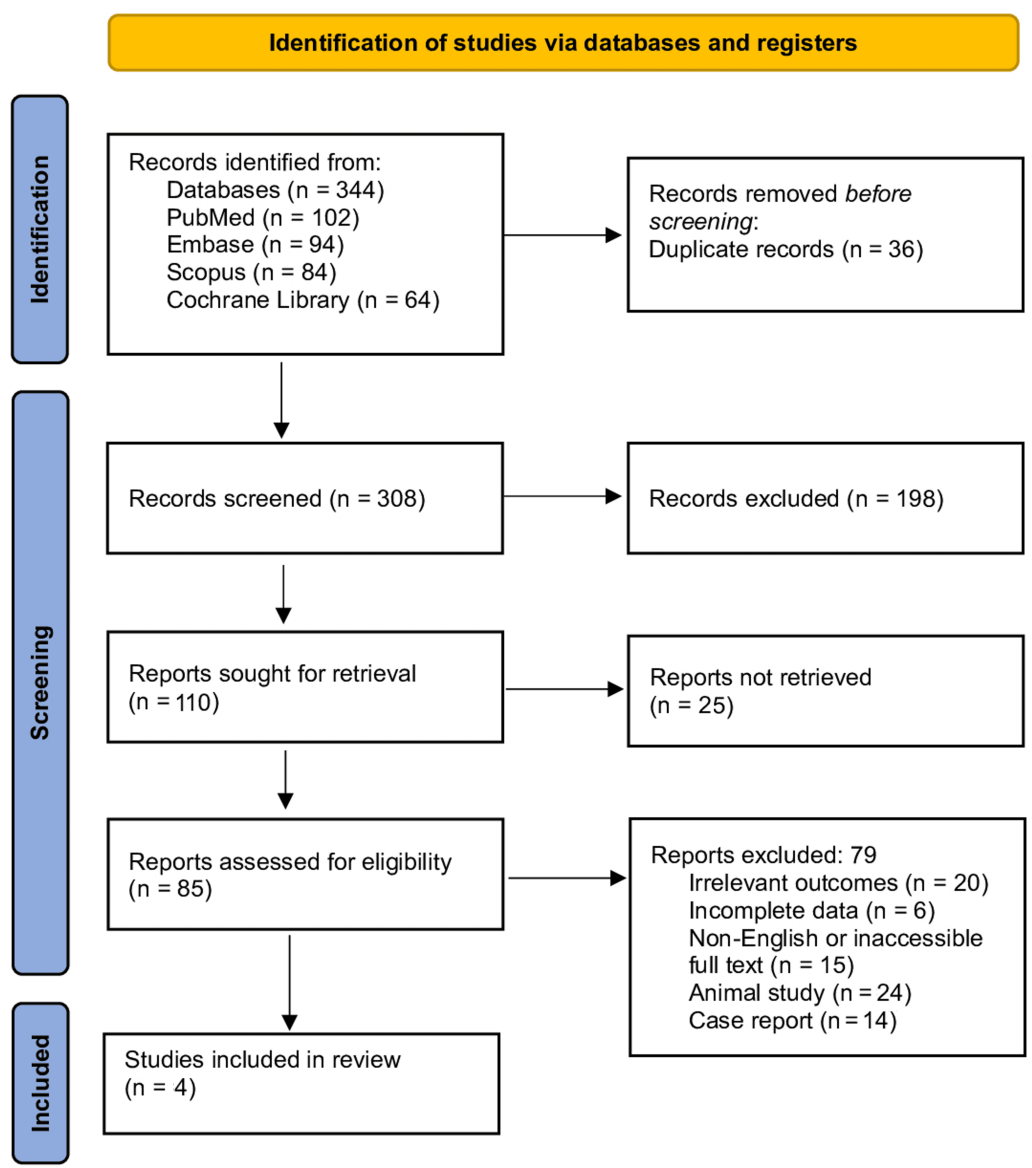
PRISMA flow diagram PRISMA: Preferred Reporting Items for Systematic reviews and Meta-Analyses.

A total of 344 records were identified through database searches, including PubMed/MEDLINE (n = 102), Embase (n = 94), Scopus (n = 84), and the Cochrane Library (n = 64). After removing 36 duplicates, 308 records were screened, of which 198 were excluded based on titles and abstracts. Following full-text assessment of 85 reports, 79 were excluded for reasons such as being case reports, editorials, conference abstracts, animal studies, or lacking defined outcomes. Ultimately, four studies were included in the final review (Figure 1). 

Characteristics of the Selected Studies

Table 1 demonstrates the findings of the included studies evaluating diverse approaches to triage in emergency and disaster settings. Bazyar et al. (2022) conducted a systematic review analyzing the accuracy of various triage systems in disasters and MCIs, highlighting the importance of adaptability to pathophysiological changes under crisis conditions [13]. Suamchaiyaphum et al. (2024) performed an evidence-based review assessing factors influencing triage accuracy among emergency nurses, identifying knowledge, experience, and situational awareness as key determinants of effective patient sorting [14]. Tahernejad et al. (2024) reviewed the role of AI in triage during emergencies and disasters, emphasizing its potential to improve accuracy, speed, and resource allocation while addressing integration and real-time adaptability challenges [15]. Gao et al. (2023) summarized evidence on the use of portable ultrasound integrated with AI at prehospital emergency and disaster sites, demonstrating enhanced diagnostic capacity and more precise triage decisions, ultimately improving survival rates and reducing delays in critical care [16]. Collectively, these studies highlight the evolving role of technology, training, and evidence-based frameworks in optimizing triage performance and patient outcomes in high-pressure environments.

**Table 1:** Characteristics of the selected studies P: Population, the group of participants or patients included in the study; I: Intervention/Exposure, the specific triage system, technology, or factor being examined; C: Comparator, the reference group or alternative approach used for comparison; O: Outcomes, the primary and secondary results measured, such as accuracy, timeliness, or survival rates; AI: Artificial intelligence, computational algorithms designed to support decision-making in triage; Prehospital: Care provided before arrival at a hospital, typically at the scene of the incident; Triage Impact: Influence of the intervention or approach on patient prioritization and management; Importance: Broader significance of the findings for clinical practice, training, or policy.

Study	Population (P)	Exposure/Condition (I)	Comparator (C)	Outcomes (O)	Pathophysiological Findings	Triage Impact	Importance
Bazyar et al. (2022) [13]	Studies involving patients in disasters and mass casualty incidents	Use of various triage systems	Alternative triage systems or no standardized system	Accuracy, timeliness, and reliability of triage	Highlights the physiological prioritization of patients based on injury severity and resource availability	Improved accuracy and reduced over-/under-triage in mass casualty events	Supports evidence-based refinement of triage protocols for disaster settings
Suamchaiyaphum et al. (2024) [14]	Emergency nurses in acute care and disaster scenarios	Factors influencing triage accuracy (training, experience, environment)	Nurses with different levels of exposure or training	Triage accuracy and decision-making speed	Shows cognitive and physiological stress affecting accuracy under high-pressure conditions	Identifies training gaps and environmental influences on correct patient prioritization	Informs targeted training and simulation-based competency development
Tahernejad et al. (2024) [15]	Studies on emergency and disaster triage	Application of AI in triage	Conventional/manual triage methods	Speed, precision, and adaptability of triage	AI enhances recognition of life-threatening conditions via algorithmic decision support	Increased precision and reduced human error in mass casualty sorting	Demonstrates AI’s role as a force multiplier in emergency response
Gao et al. (2023) [16]	Prehospital emergency and disaster scene	Portable ultrasound combined with AI	Standard assessment without ultrasound-AI integration	Early diagnosis, triage accuracy, and intervention timeliness	Detects internal injuries and critical pathophysiological changes prehospital	Allows faster critical care prioritization at the scene	Enhances on-site diagnostic capability and patient survival chances

Risk of Bias Assessment

Table 2 presents the risk of bias assessment for the included studies. Bazyar et al. (2022) and Tahernejad et al. (2024), both systematic reviews, were assessed using the AMSTAR-2 tool, showing moderate risk due to limited reporting of excluded studies. Suamchaiyaphum et al. (2024), an evidence-based review, was also assessed using the AMSTAR-2 tool, which rated moderate risk for unclear handling of confounders. Gao et al. (2023), a narrative review, was evaluated using the SANRA scale, showing low risk with good methodological clarity but limited critical appraisal of included sources. 

**Table 2:** Risk of bias assessment AMSTAR-2: A Measurement Tool to Assess Systematic Reviews version 2, a validated critical appraisal tool for systematic reviews; SANRA: Scale for the Assessment of Narrative Review Articles, a tool used to evaluate the quality of narrative reviews.

Study	Study Design	Risk of Bias Tool	Risk of Bias Rating	Justification
Bazyar et al. (2022) [13]	Systematic review	AMSTAR-2	Moderate	Comprehensive literature search and inclusion of multiple databases.
Suamchaiyaphum et al. (2024) [14]	Evidence-based review	AMSTAR-2	Moderate	Used clear inclusion/exclusion criteria and systematic data extraction; however, limited details on risk of bias in included studies and absence of meta-analysis affected methodological strength.
Tahernejad et al. (2024) [15]	Systematic review	AMSTAR-2	Low	Search strategy transparent, robust risk of bias assessment conducted for included studies, and results synthesized comprehensively with minimal methodological limitations.
Gao et al. (2023) [16]	Narrative review	SANRA	High	Provided descriptive synthesis and relevant context but lacked a systematic search strategy, formal bias assessment, and clear inclusion criteria, increasing the risk of selection and reporting bias.

Discussion

In mass disasters and large-scale emergencies, triage remains the cornerstone of effective prehospital and hospital response, determining not only the prioritization of patients but also the eventual outcomes. The process begins at the scene, where first responders rapidly assess airway, breathing, circulation, neurological status, and mobility, applying systems such as START, JumpSTART, SALT, or MPTT [17]. The fundamental principle of triage is to allocate limited medical resources to those most likely to benefit, thereby maximizing survival. However, this process is complex, and errors in accuracy or delays in timeliness may lead to both over-triage, straining limited resources, and under-triage, resulting in preventable mortality.

Several studies have investigated the accuracy and timeliness of triage in MCIs, offering insights into the physiological, cognitive, and systemic factors that influence its effectiveness. For instance, Bazyar et al. (2022) demonstrated that structured triage systems significantly reduce over- and under-triage rates compared to ad hoc decision-making, thus supporting evidence-based refinements to triage protocols in disaster medicine [13]. Similarly, Suamchaiyaphum et al. (2024) focused on the human element, showing how nurse training, clinical experience, and environmental pressures directly influence the speed and accuracy of decision-making under stressful conditions [14]. These findings highlight that while triage tools provide structured algorithms, the cognitive load and stress response of responders also play a pivotal role in determining outcomes.

Recent advancements in technology have added a new dimension to the accuracy and speed of triage. Tahernejad et al. (2024) examined the role of AI in disaster triage and reported that AI-supported systems reduce human error by providing real-time algorithmic decision support for classifying patients, particularly in high-volume casualty incidents [15]. Similarly, Gao et al. (2023) explored the integration of portable ultrasound with AI-based algorithms for prehospital triage, demonstrating that such innovations enable early recognition of occult internal injuries and guide faster initiation of life-saving interventions [16]. These technological tools enhance precision and timeliness, acting as force multipliers that augment the responder’s clinical judgment.

The time factor remains central to the effectiveness of triage. Even a few minutes of delay in categorizing critically injured patients can drastically affect morbidity and mortality. Evidence shows that structured tools like MPTT outperform conventional systems in rapidly identifying patients who require urgent interventions, thereby improving survival rates in mass casualty events. Furthermore, the transition from prehospital to in-hospital triage is equally vital. On arrival, patients are reassessed under hospital-based triage systems such as ESI, Manchester Triage System (MTS), or Canadian Triage and Acuity Scale (CTAS) [18]. These tools stratify patients not only by injury severity but also by expected resource utilization, ensuring that diagnostic and surgical resources are efficiently allocated. In their systematic review, Zakeri et al. reported that re-triage within the hospital setting corrected a significant proportion of misclassifications from the prehospital phase, underscoring the importance of layered triage strategies [19].

Ultimately, the literature converges on the principle that both accuracy and timeliness of triage profoundly influence patient outcomes in disaster medicine. While traditional systems remain valuable, human factors such as stress, training gaps, and environmental chaos can compromise effectiveness. The incorporation of technological tools such as AI and portable imaging has shown promise in addressing these limitations. However, challenges remain, including ensuring universal training, maintaining inter-system consistency, and adapting algorithms to the unpredictable realities of mass disasters. Limitations of the current evidence include heterogeneity of study designs, simulation-based methodologies rather than real disaster validation, and limited data from low-resource settings where triage is often most critical. Most comparative data derive from simulations, retrospective registries, or single-event reports rather than prospective multicenter evaluations during true large-scale disasters, limiting external validity. Additionally, studies are concentrated in high-income settings and often use heterogeneous outcome definitions, reducing generalizability to low-resource contexts and complicating pooled interpretation.

## Conclusions

Prehospital triage in MCIs is a critical process aimed at balancing accuracy and timeliness to optimize survival and resource allocation. Traditional systems such as START, JumpSTART, SALT, CareFlight, and Triage Sieve remain widely used, though each demonstrates variable sensitivity, specificity, and risks of over- or under-triage. Recent advances, including physiology-based protocols like MPTT and technology-assisted approaches using AI and portable diagnostics, show promise in improving both speed and precision. Training, responder experience, and situational awareness remain pivotal factors influencing real-world performance, underscoring the need for ongoing education and simulation-based preparedness. Current evidence, however, is limited by heterogeneity, reliance on simulation data, and underrepresentation of low-resource contexts, highlighting the urgent need for prospective multicenter evaluations during actual disasters. Ultimately, no single triage algorithm is universally applicable, and optimizing outcomes will require a combination of evidence-based refinement, technological innovation, and context-specific adaptability.
